# Effect of the exercise of walkers performed in public squares with spontaneous or prescribed intensity on post-exercise hypotension

**DOI:** 10.1590/S1518-8787.2017051006247

**Published:** 2017-07-07

**Authors:** Taís Feitosa da Silva, Alesandra Araújo de Souza, Fabiano Ferreira de Lima, Jennifer Ariely Sales Suassuna, Henrique Eduardo Paiva Lira do Couto, Gustavo Roque Tenório, Maria Irene de Andrade Gomes Silva, Guilherme Leandebal Bonifácio Dias, Alexandre Sérgio Silva

**Affiliations:** I Departamento de Educação Física. Universidade Federal da Paraíba. João Pessoa, PB, Brasil; II Programa Associado de Pós-Graduação em Educação Física. Universidade de Pernambuco. Universidade Federal da Paraíba. Recife, PE, Brasil

**Keywords:** Walking, Post-Exercise Hypotension, Hypertension

## Abstract

**OBJECTIVE:**

To quantify the intensity adopted by walkers in public squares and check the occurrence and magnitude of post-exercise hypotension in the spontaneously adopted intensity and in a prescribed intensity.

**METHODS:**

In 98 volunteers (38 of them being hypertensive), walkers in public squares of the city of João Pessoa, State of Paraíba, Brazil, we have identified the intensity of a usual training monitored by heart rate and we have investigated the occurrence and magnitude of post-exercise hypotension. Subsequently, participants were instructed to walk with moderate intensity. Blood pressure was measured after rest and during post-exercise recovery.

**RESULTS:**

Of the total participants, 41% of the hypertensive and 36% of the normotensive individuals walked with light intensity. With the prescription, intensity increased to 55% and 52%, for the hypertensive and normotensive individuals, respectively. In the usual and prescribed intensity, the hypertensive individuals had post-exercise hypotension of -3.7±11.6 mmHg and -4.72±12.8 mmHg, respectively. There was no correlation between post-exercise hypotension and the initial systolic component of the hypertensive individuals (r^2^ = 0.2; p < 0.002).

**CONCLUSIONS:**

Walkers in public squares choose light intensity for walking. When they exercise with the prescribed intensity, they increase the intensity, but the magnitude of the PEH is not increase with this guidance.

## RESUMO

**OBJETIVO:**

Quantificar a intensidade adotada por caminhantes em praças públicas e verificar a ocorrência e a magnitude da hipotensão pós-exercício na intensidade espontaneamente adotada e em uma intensidade prescrita.

**MÉTODOS:**

Em 98 voluntários (38 hipertensos), caminhantes em praças públicas da cidade de João Pessoa, PB, identificamos a intensidade de um treino habitual monitorada por meio da frequência cardíaca e averiguamos a ocorrência e magnitude de hipotensão pós-exercício. Posteriormente, os participantes foram instruídos a caminhar com intensidade moderada. A pressão arterial foi aferida após o repouso e durante a recuperação pós-exercício.

**RESULTADOS:**

Do total de participantes, 41% dos hipertensos e 36% dos normotensos caminhavam com intensidade leve. Com a prescrição, a intensidade aumentou para 55% e 52%, para hipertensos e normotensos. Na intensidade habitual e prescrita, os hipertensos obtiveram hipotensão pós-exercício de -3,7±11,6 mmHg e -4,72±12,8 mmHg. Houve correlação entre hipotensão pós-exercício e o componente sistólico inicial dos hipertensos (r^2^ = 0,2; p < 0,002).

**CONCLUSÕES:**

Caminhantes em praças públicas selecionam intensidade leve para realização de caminhada. Quando realizam exercício com intensidade prescrita, aumentam discretamente a intensidade, mas não obtêm aumento da magnitude da HPE com esta orientação.

## INTRODUCTION

Systemic hypertension is the most prevalent of the chronic degenerative diseases. This public health problem is aggravated by the difficulty in controlling blood pressure (BP), especially when treated with only monotherapy^1^ ^,^ ^2^. To increase the possibilities of control, the combination of drugs with non-drug therapy is indicated^3^ ^,^ ^4^.

Physical exercise is one of the most effective non-pharmacological tools for the treatment of hypertension. In a systematic review^5^, it has been noted that hypertensive persons have a clinical and ambulatory acute hypotensive response to aerobic exercise sessions. These reductions become relevant as they can last up to 22 hours^6^ and are called post-exercise hypotension (PEH)^7^.

However, the effectiveness of exercise in the treatment of hypertension is controversial. It is known that 75% of hypertensive individuals can reduce their BP, but these data refer to a chronic reduction in response to aerobic exercise training programs^8^. Moreover, studies that have been conducted to quantify the percentage of hypertensive persons responsive or resistant to reduction of post-exercise blood pressure (BP) are nonexistent. The intensity, duration, and type of exercise affect the magnitude of the blood pressure response^5^ ^,^ ^9^, and they could explain at least partially the variability in blood pressure reduction with exercise sessions or training programs. Higher values of PEH are obtained with aerobic exercises, with a duration of 30 min to 60 min^6^ ^,^ ^9^. However, the intensity is considered the most determining variable – better values of PEH have been found after exercises performed with light and moderate intensity^7^ ^,^ ^9^, which corresponds to 60%–85% of maximum heart rate.

However, these data are from models under laboratory conditions. In public squares, these variables are not controlled. In fact, we have empirically observed that practitioners of exercises in public squares do so with intensity below the recommended one. These empirical observations are not documented in the literature, so that there are no intensity data adopted by practitioners of exercises when they realize their activities without professional guidance. The knowledge on the intensity adopted by practitioners of exercise in public squares is relevant to investigate whether the responses of laboratory studies are confirmed in the practical reality and to check the need of these practitioners to be guided to obtain the best benefits of the physical training.

In this study, we have tested two hypotheses: 1) walkers or runners in public squares without professional guidance have training sessions with intensity below what is proposed in the literature as the ideal to promote PEH and 2) the execution of a session with prescribed intensity improves the magnitude of the PEH.

Therefore, we have determined the intensity adopted by hypertensive and normotensive individuals who run or walk in public squares and we have checked the occurrence and magnitude of PEH in the intensity usually adopted. Additionally, we have tested whether the performance of the exercise with the intensity proposed in the literature increased the magnitude of the PEH.

## METHODS

This is a population-based, representative, and cross-sectional study. For sample calculation, we considered that 20% of a total of 452,000 hypertensive persons of a Brazilian State capital exercised enough for health promotion (above 1,000 kcal per week)^10^. Adopting a sampling error of 5% and a confidence level of 90%, we found a sample size of 98 volunteers for the study. We selected a public square used for walking and running by the population of each of the five health districts that geographically divide the city by the network of health services. From each health district, we intentionally choose the public square recognized by the researchers as the most frequented for walking and running.

To participate in the study, volunteers should: 1) have been walking or running for at least three months, 2) have no joint damage that would hinder the practice of the exercise, and 3) be aged between 30 and 60 years.

The researchers positioned themselves in a place considered as the point of arrival in that environment and approached all the volunteers, except for children and adolescents, because they were clearly outside the age group under study. We approached 161 volunteers. After screening based on the inclusion criteria and considering the sample losses, the sample amounted to 98 volunteers (44.8 [SD = 8.9] years), being 38 men (43.1 [SD = 8.5] years) and 60 women (46.1 [SD = 9] years), of which 38 were hypertensive and 60 were normotensive practitioners of physical exercise in public squares of the city of João Pessoa, state of Paraíba, Brazil. Participants were chosen at random, approached in the squares where the research was conducted.

The volunteers carried out two sessions of aerobic exercise, walking or running, for 30 minutes with at least an interval of 48 hours between them. In the first session, participants were instructed to perform the exercise in their usual way, in terms of speed adopted to walk or run. In the second session, the intensity was previously prescribed by the researchers within the parameters suggested in the literature for aerobic exercises. Heart rate (HR) and subjective perception of effort (SPE) were recorded during the exercises. The BP was measured at rest and for 30 minutes after each exercise session. For the two experimental sessions, volunteers should not practice physical exercises for 48 hours.

To monitor the exercise performed in the usual intensity, we measured HR after a period of rest before the exercise and every 7’30” during the 30 minutes of exercise. This time was adopted as it allows for an appropriate monitoring of the exercise intensity without frequently interrupting for measurement. The HR was monitored by the palpatory method in the radial artery for 15 seconds; the interruption of the exercise for this measurement lasted less than 30 seconds. The researchers were experienced and skilled in the technique adopted to measure the HR. Before the exercise, the volunteers were instructed to use the scale of subjective perception of effort of Borg from six to 20^11^. The records were strictly carried out at the end of each measure of HR.

The monitoring of the exercise performed at the prescribed intensity was done at least 48 hours after the session with usual intensity. The intensity of the exercise was prescribed based on what is recommended by the American College of Sports Medicine^12^, which proposes that aerobic exercises should be practiced with a HR between 60% and 85% for normotensive individuals and between 60% and 80% of maximum heart rate (HRmax) for hypertensive individuals. The calculations were based on the equation proposed by Karvonen et al.^13^ The HRmax was estimated using the following equation: HRmax = 220 - age of the volunteer. The values of HR corresponding to 60% and 80% of the HRmax reserve were presented before the exercise session and the volunteers were instructed to maintain a speed that would result in a HR within the range shown. Every 7’30”, the volunteers were informed of the HR and asked to adjust their speed if the values were outside of the range informed prior to the exercise. At the same time, they reported their subjective perceptions of effort. The procedures to measure the HR and SPE were the same as in the session with usual intensity. The BP measurements were performed by the auscultatory method following the V Diretrizes Brasileiras de Hipertensão Arterial (V Brazilian Hypertension Guidelines)^14^. The volunteers were asked to remain seated for 10 minutes, and we measured the resting HR at the end of this period. After the end of the exercise, the volunteers were asked again to remain seated for 30 minutes, for recovery. We measured BP immediately after the exercise and every 10 minutes during the period of recovery.

The data are expressed as average and standard deviation and as percentages of descriptive statistics. We used the normality and homogeneity test (Kolmogorov-Smirnov) in all of the data. Possible differences between the behavior of BP, HR, and SPE, intra- and inter-procedure, were tested using two-way ANOVA with Tukey’s test. Pearson correlation was used between the magnitude of the PEH and the socio-demographic variables (age and gender) and BMI. We adopted a confidence level of 5%.

This study has been approved by the research ethics committee with human beings of the Hospital Universitário Lauro Wanderley (Protocol 625/10). All volunteers signed the informed consent according to Resolution 196/96 of the National Health Council.

## RESULTS

In this study, we have found prevalence of 38.8% of hypertensive individuals (47.9±9.42 years) between walkers and runners in public squares of the city of João Pessoa, State of Paraíba, Brazil (Table). Among the hypertensive individuals, 63.2% (n = 14) of them had controlled BP (138 mm Hg [SD = 8] and 90 mmHg [SD = 5] for systolic and diastolic, respectively), and 36.8% of them had decompensated values (138 mm Hg [SD = 8] and 90 mmHg [SD = 5] for systolic and diastolic, respectively).

**Table:** Characterization of the volunteers of the study according to presence of declared or diagnosed hypertension, age, body mass index, initial systolic and diastolic blood pressure, and resting heart rate.

	General		Hypertensive		Normotensive		p
		
	Average	SD	Average	SD	Average	SD	
Frequency*	98	100	38	38.8	60	62.2	
Ratio (women/men)	58/40	-	20/18	-	38/22	-	
Age (years)	45	9	48	10	43	8	**0.027**
Body mass index (kg/m^2^)	28	5	29.3	8	29.9	4	**0.002**
Systolic blood pressure (mmHg)	115	16	122	16	111	14	**0.003**
Diastolic blood pressure (mmHg)	79	10	83	9	76	9	**0.002**
Heart rate (bpm)	80	11	80	9	80	11	0.999

* n and percentage.

Significant difference between hypertensive and normotensive individuals presented in bold.

**Figure 1:**
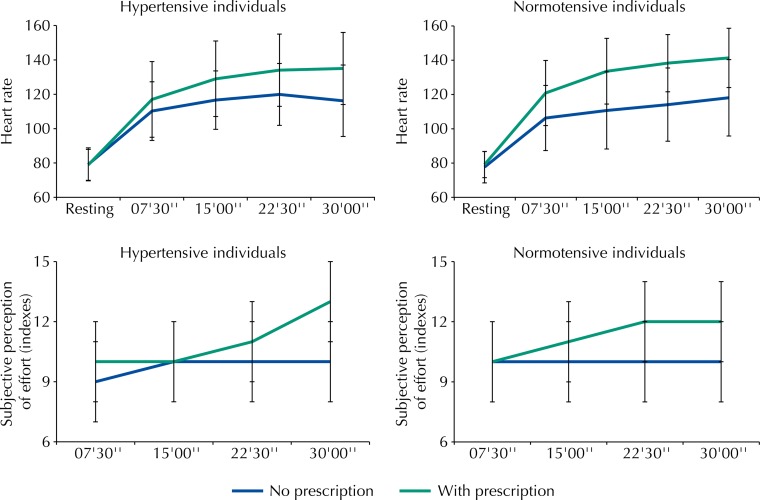
Behavior of the heart rate and subjective perception of effort in hypertensive and normotensive individuals on days with and without exercise prescription.

Figure 1 shows the behavior of the HR and the SPE reported by the hypertensive and normotensive individuals in the exercises performed with usual and previously prescribed intensity. When the volunteers performed the exercise in the usual intensity, both the hypertensive and the normotensive individuals kept HR below the limits set for the target area: at 07’30”, 15’00”, 22’30”, 30’00” of the exercise, the HR corresponded to, respectively, 36%, 42%, 45%, and 41% of HRmax for the hypertensive individuals (average intensity of 41%) and 31%, 36%, 38%, and 42% for the normotensive individuals (average intensity of 36%).

The lines parallel to the x-axis represent the limits established for the target zone of the training and the subjective perception of effort. The lines parallel to the y-axis represent the lower and upper limits of heart rate and subjective perception of effort.

When instructed to perform the exercise with the previously prescribed intensity, the hypertensive individuals succeeded in complying with this guidance at 22’30” and 30’00”. They reached 40%, 56%, 60%, and 61% of HRmax when the HR was measured (average of 52%). For this intensity of exercise, the hypertensive individuals increased the SPE to values between 10 and 11. The normotensive individuals approached the target established at 7’30” and reached the guidance in the following measurements (42%, 55%, 60%, and 63% of HRCmax), with an average of 55%. For this procedure, they reported SPE between 10 and 11 (average intensity of 10.5%).

**Figure 2:**
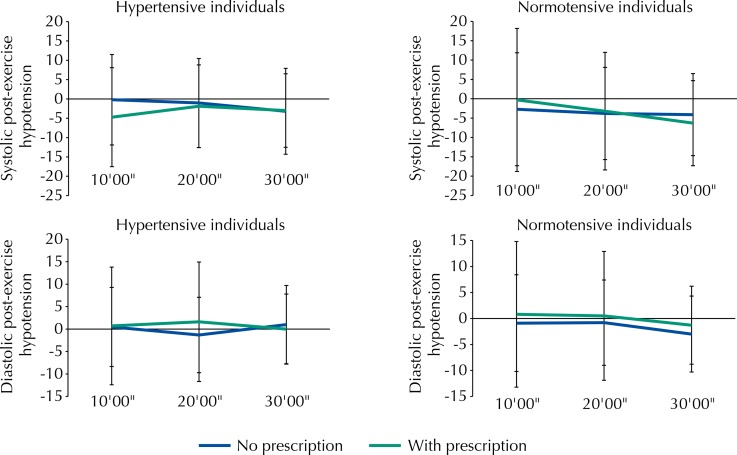
Magnitude of the systolic and diastolic hypotension of hypertensive and normotensive individuals at 10’00”, 20’00”, and 30’00” of post-exercise recovery. No significant differences were found between the moments of measurement of blood pressure of the hypertensive and normotensive individuals for p < 0.05.

In the exercise performed with the usually adopted intensity, the hypertensive individuals obtained PEH of -0.21 mmHg [SD = 11.7] and 0.47 mmHg [SD = 8.8] at 10’, -1.05 mmHg [SD = 11.5] and -1.32 mmHg [SD = 8.4] at 20’, and -3.7 mmHg [SD = 11.6] and -1.6 mmHg [SD = 8.5] at 30’ of recovery for systolic and diastolic pressure, respectively. The magnitude of the PEH was kept when they exercised with the previously prescribed intensity (-4.72 mmHg [SD = 12.8] and 0.70 mmHg [SD = 13.1] at 10’, -1.89 mmHg [SD = 10.7] and 1.62 mmHg [SD = 13.3] at 20’, and -3.0 mmHg [SD = 9.5] and 0.0 mmHg [SD = 7.8] at 30’ for SBP and DBP, respectively) (Figure 2).

In the exercise performed with the usually adopted intensity, the normotensive individuals obtained PEH of -2.7 mmHg [SD = 14.6] and -0.9 mmHg [SD = 9.3] at 10’, -3.8 mmHg [SD = 11.9] and -0.8 mmHg [SD = 8.2] at 20’, and -4.1 mmHg [SD = 10.6] and -3 mmHg [SD = 7.3] at 30’ of recovery for systolic and diastolic pressure, respectively. The magnitude of the PEH was kept when they exercised with the previously prescribed intensity (-0.3 mmHg [SD = 18.5] and 0.8 mmHg [SD = 14] at 10’, -3.2 mmHg [SD = 15.2] and 0.5 mmHg [SD = 12.4] at 20’, and -6.3 mmHg [SD = 11] and 1.3 mmHg [SD = 7.5] at 30’ for SBP and DBP, respectively) (Figure 2).

**Figure 3:**
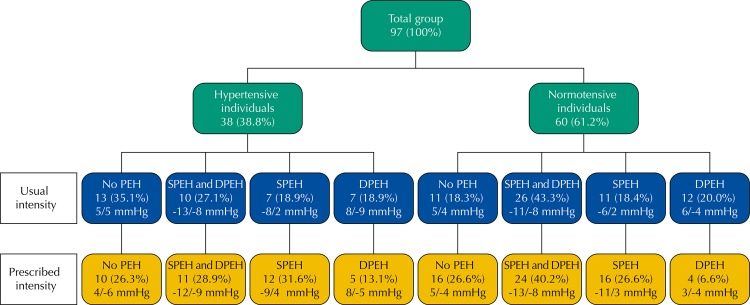
Percentage of hypertensive and normotensive individuals who presented concomitant, systolic, and diastolic PEH or those who did not obtain it in the usual intensity (top line) and in the prescribed intensity (bottom line), with average of the values of the systolic and diastolic PEH, respectively.

PEH: post-exercise hypotension; SPEH: systolic post-exercise hypotension; DPEH: diastolic post-exercise hypotension

Among the hypertensive individuals, 27% presented both systolic and diastolic PEH in the exercise performed in the usual intensity. The performance of the prescribed intensity exercise only slightly increased the percentage of systolic and diastolic reduction, concomitantly. Among the normotensive individuals, 43.3% had systolic and diastolic hypotension at the same time in the moderate intensity exercise, and the prescription resulted in discreet reduction of this value. The data showing the percentage of the systolic and diastolic reduction in isolation are presented in Figure 3. We can observe that exercise following a prescription with increased intensity does not increase the percentage of hypertensive individuals who obtain success in reducing post-exercise blood pressure.

**Figure 4:**
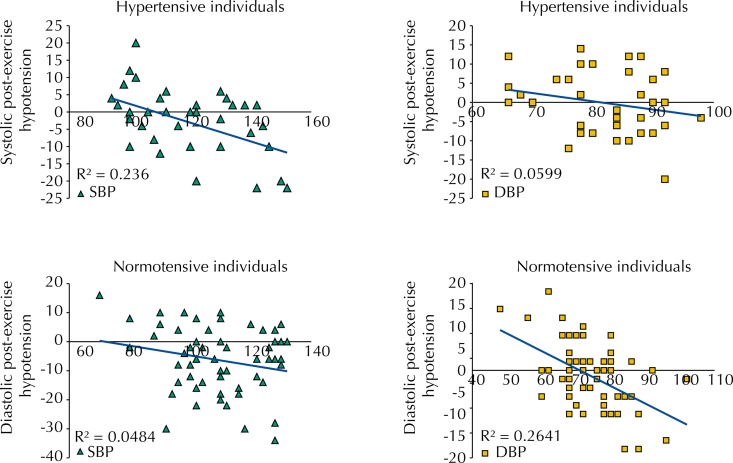
Correlation of the magnitude of systolic and diastolic PEH with initial blood pressure level of hypertensive and normotensive individuals on the day with prescribed exercise. Statistical difference was found for p < 0.05.

SBP: systolic blood pressure; DBP: diastolic blood pressure; PEH: post-exercise hypotension

Based on the data of the exercise performed with previously prescribed intensity, correlation tests show that the initial BP level was a variable that influenced PEH. Among the hypertensive individuals, higher values of systolic blood pressure in the moments before exercise showed a positive correlation with the magnitude of the PEH, which did not happen for the diastolic blood pressure. Among the normotensive individuals, this correlation only happened for the diastolic component (Figure 4).

On the other hand, the variables age and BMI did not show correlations with the magnitude of the PEH, neither for the hypertensive or normotensive individuals. Among the hypertensive individuals, the correlation between age and PEH resulted in r^2^ = 0.006 (p = 0.70) for systolic PEH and r^2^ = 0.001 (p = 0.87) for diastolic PEH. Among the normotensive individuals, we found r^2^ = 0.02 (p = 0.44) for systolic PEH and r^2^ = 0.002 (p = 0.81) for diastolic PEH. The correlation between BMI and systolic PEH resulted in r^2^ = 0.04 (p = 0.34) for systolic PEH and r^2^ = 0.05 (p = 0.32) for diastolic PEH. And for the normotensive individuals, we found r^2^ = 0.04 (p = 0.34) for systolic PEH and r^2^ = 0.00 (p = 0.97) for diastolic PEH. The correlation between initial systolic BP and PEH for the hypertensive individuals resulted in r^2^ = 0.24 (p = 0.002), and for the normotensive individuals it resulted in r^2^ = 0.03 (p = 0.19). Regarding initial diastolic BP, for the hypertensive individuals it resulted in r^2^ = 0.06 (p = 0.14) and for the normotensive individuals it resulted in r^2^ = 0.26 (p < 0.001).

## DISCUSSION

The main findings of this study were: 1) both hypertensive and normotensive individuals usually walk with intensity below the target area; 2) when encouraged to walk in the target area, the normotensive individuals succeeded, but the hypertensive individuals only managed to do so at the end of the exercise; 3) even when below the target area, the hypertensive and normotensive individuals obtain PEH; 4) PEH does not increase when the hypertensive or normotensive individuals are instructed to exercise with greater intensity than what they are used to; 5) the only variable that influenced PEH was initial BP level.

When asked to exercise within the target area prescribed by the researchers, the volunteers increased the intensity of the exercise, but the hypertensive individuals only reached the prescribed intensity at the end of the exercise and the normotensive individuals did so only at the final third of the session. This phenomenon can be explained by a behavioral trend evidenced by the data of a relatively new line of research, which has indicated a preference of persons in practicing exercises with self-selected intensity, over a prescribed intensity^15^ ^,^ ^16^. This self-selected intensity was lower than what is classically recommended as ideal to provide the benefits of aerobic exercise^12^.

A peculiarity of this study was that it was conducted in public squares, in the same environmental conditions in which subjects practice their exercises. The exercise performed under laboratory conditions and with the use of treadmills or cycle ergometers would lead to greater success in achieving the recommended exercise intensity. However, our data highlights the preference of individuals to walk in intensity below the recommended one and a resistance to increase the intensity until the level of effort prescribed, even when stimulated by the researchers.

Although the average intensity of the exercise in the usual level and the guided level among the hypertensive individuals had increased from 41% to 54% of maximum heart rate, the magnitude of the PEH did not change. The same occurred with the normotensive individuals, even though they reached the intensity close to the recommended values.

The volunteers did not reach the intensity prescribed by the researchers, what does not allow us to answer one of the questions of this study (if the exercise carried out within the recommended intensity would result in greater magnitude of PEH than the one performed in lower intensities). On the other hand, there are reports in the literature indicating a possible effectiveness of exercise performed with lower intensity to reduce BP. In fact, there are authors who argue that there is not a consensus regarding the intensity (low, moderate, or high) and the reduction of BP after exercise^5^. Low to moderate intensity exercise (40% to 60%) can be as effective as higher intensity training for hypertensive individuals^8^. This intensity range between 40% and 60% was the one adopted by volunteers of this study, both when exercising with the intensity spontaneously chosen and after being asked to adopt a previously prescribed intensity.

A preliminary literature review has indicated that 25% of hypertensive individuals are resistant to reduction of blood pressure induced by aerobic exercises^8^. However, this data refers to the chronic reduction of BP (in response to a training program of several weeks). As far as we know, there are no studies that quantify the percentage of persons responsive or resistant to PEH. Our data indicate a percentage of 35% of hypertensive individuals and 18% of normotensive individuals resistant to PEH when exercising in public squares with spontaneously selected intensity. However, when asked to exercise as previously prescribed, they did so with greater intensity and this percentage reduced to 26% in the hypertensive individuals.

The results of this study should be viewed with caution when considering the failure of the volunteers in increasing the intensity when prescribed. Thus, the percentage of hypertensive individuals resistant to PEH found in this study must be considered for an average intensity of 54% and 55% of HRmax reserve that was adopted by the hypertensive and normotensive individuals, respectively, when exercising with the intensity prescribed.

The magnitude of the PEH can be determined by circumstances related to exercise protocol (intensity, duration, type)^7^ ^,^ ^9^ ^,^ ^17^ and initial blood pressure levels (before the exercise session)^7^. Individual factors such BMI and metabolic conditions could influence the magnitude of the PEH. For example, Viegas et al.^18^ have shown that obese hypertensive individuals had reduced PEH in relation to eutrophic hypertensive individuals, but these data are limited because of the small sample (n = 16, only). Meanwhile Karavelioglu et al.^19^ have observed significantly higher values of 24-hour systolic BP in diabetic individuals who were tested in a treadmill.

Based on these data, we tested the hypothesis that age, BMI, and initial BP level could influence PEH. We observe that only the last variable really influences PEH, what has already been described previously^7^ and which was not corroborated by Viegas et al.^18^, who has shown negative association between obesity and PEH. Although we could not find an association for the variables analyzed, vasoconstricting conditions (such as an unfavorable inflammatory condition and oxidative stress) can influence PEH, so that they still need to be investigated.

In this study, PEH was monitored only until 30 minutes after the end of the exercise, while several studies monitor the PEH for at least 60 minutes^20^ ^,^ ^21^. Although the 30-minute monitoring has been sufficient to show that the intensity of the exercise and the way of doing it (self-monitored or prescribed) does not modify PEH, the magnitude of this phenomenon could be different given an increased monitoring after the exercise. Another limitation was the lack of randomization between the sessions with usual and prescribed intensity. However, this model was necessary because the researchers would certainly influence the speed usually adopted if the sessions were randomized. An alternative would be a pilot study to determine the usually adopted intensity and then use it and randomize the sessions. However, with this procedure, we would lose the actual circumstances in which individuals perform their exercises in public environments. The research was developed in an external environment and we monitored up 10 to 12 persons per day, which hindered the use of frequency meters, as these instruments exist in a limited number in the environment where the research was conducted. Thus, we opted for the palpatory method to measure HR, as it offers an acceptable alternative to measure auscultatory systolic BP in clinical practice, as proposed by Van Der Hoeven et al.^22^

Considering the set of data obtained and the limitations pointed out, we verify that an important practical implication of this study is that we must consider the greater tendency of individuals to exercise lightly and with self-selected intensity. The increase of the intensity after the guidance of the researchers does not increase the average magnitude of the PEH, which may indicate that individuals can be encouraged to keep their walking in public squares by adopting a self-selected intensity. However, the increase in the percentage of persons who have a reduction in blood pressure after exercising shows the need to guide the hypertensive individuals who perceive the absence of PEH after their walking sessions. After testing a few conditions that could influence PEH, we have found that only initial BP is associated with the reduction of blood pressure induced by exercise.
